# Vanin-1 Pantetheinase Drives Smooth Muscle Cell Activation in Post-Arterial Injury Neointimal Hyperplasia

**DOI:** 10.1371/journal.pone.0039106

**Published:** 2012-06-13

**Authors:** K. Jagadeesha Dammanahalli, Stephanie Stevens, Robert Terkeltaub

**Affiliations:** San Diego VA Healthcare System and Department of Medicine, University of California San Diego, San Diego, California, United States of America; Medical University Innsbruck, Austria

## Abstract

The pantetheinase vanin-1 generates cysteamine, which inhibits reduced glutathione (GSH) synthesis. Vanin-1 promotes inflammation and tissue injury partly by inducing oxidative stress, and partly by peroxisome proliferator-activated receptor gamma (PPARγ) expression. Vascular smooth muscle cells (SMCs) contribute to neointimal hyperplasia in response to injury, by multiple mechanisms including modulation of oxidative stress and PPARγ. Therefore, we tested the hypothesis that vanin-1 drives SMC activation and neointimal hyperplasia. We studied reactive oxygen species (ROS) generation and functional responses to platelet-derived growth factor (PDGF) and the pro-oxidant diamide in cultured mouse aortic SMCs, and also assessed neointima formation after carotid artery ligation in vanin-1 deficiency. *Vnn1* ^−*/*−^ SMCs demonstrated decreased oxidative stress, proliferation, migration, and matrix metalloproteinase 9 (MMP-9) activity in response to PDGF and/or diamide, with the effects on proliferation linked, in these studies, to both increased GSH levels and PPARγ expression. *Vnn1^−/−^* mice displayed markedly decreased neointima formation in response to carotid artery ligation, including decreased intima:media ratio and cross-sectional area of the neointima. We conclude that vanin-1, via dual modulation of GSH and PPARγ, critically regulates the activation of cultured SMCs and development of neointimal hyperplasia in response to carotid artery ligation. Vanin-1 is a novel potential therapeutic target for neointimal hyperplasia following revascularization.

## Introduction

SMC proliferation, MMP activation, and migration play pivotal roles in the progression of intimal lesions in atherosclerosis, and in arterial injury following interventional revascularization [1]. There are multiple mediators of intimal proliferation and neointima formation in model arterial injury, with some evidence suggesting that reactive oxygen species (ROS) generation and oxidative stress are a common thread [2]. In this light, reduced glutathione (GSH) is the major cellular thiol, and increasing cellular GSH levels can be protective against oxidative stress [3]. GSH homeostasis is regulated by oxidation of GSH to glutathione disulfide (GSSG), with GSH subsequently regenerated by glutathione reductase, expressed under the control of antioxidant response elements [4], [5]. In addition, cell and tissue levels of GSH stores are regulated by gammaglutamylcysteine synthetase (γGCS), the rate-limiting enzyme for GSH synthesis [6].

Vanin-1 is a widely expressed glycosyl-phosphatidylinositol (GPI)-linked plasma membrane pantetheinase ectoenzyme, and nitrilase superfamily member, that contains the invariant catalytic triad residues glutamate, lysine, and cysteine [7], [8]. Vanin pantetheinase activity generates the amino-thiol cysteamine, and regulates a variety of responses to stress [9], [10]. Cysteamine inhibits γGCS [5]. Concordantly, GSH stores in multiple tissues are significantly increased in vanin-1 knockout mice [10]. Cysteamine also appears to decrease protective activities of superoxide dismutase (SOD) and glutathione peroxidase (GSH-Px) against ROS toxicity, allowing free radical production to overwhelm antioxidant defense systems [11]. Conversely, vanin-1 deficiency is associated with both decreased superoxide production, and resistance to oxidative stress and tissue injury induced *in vivo* by paraquet or γ-irradiation [5], [10], [12].

Several inflammatory stimuli, exemplified by PDGF, promote SMC proliferation and migration [13], [14]. Vanin-1, like several other mediators of oxidative stress, promotes inflammation [15], [16]. For example, in intestinal epithelial cells, vanin-1 facilitates expression of certain pro-inflammatory cytokines [9]. Conversely, vanin-1 deficiency is protective *in vivo* for intestinal inflammation and injury in response to both nonsteroidal anti-inflammatory drugs and Schistosomal infection *in vivo* [17], [18].

Vanin-1 promotes inflammation partly by suppression of both PPARγ expression and PPARγ signal transduction [15]. PPARγ functions as an anti-inflammatory checkpoint in multiple inflammatory settings, and in a variety of cell types [18]. PPARγ-activating thiazolidinedione (TZD) treatment suppresses SMC proliferation and migration *in vitro,* as well as *in vivo* in the intimal hyperplasia process after arterial injury [18].

Furthermore, PPARγ is expressed by normal vascular SMCs, and arterial PPARγ expression normally increases in early human atherosclerotic lesions and 1–2 weeks after arterial balloon injury in rodents [19], [20]. PPARγ activation inhibits SMC invasion, and suppresses induction by PDGF of the transcription factor Ets-1, a mediator of MMP expression and SMC invasion both in cultured SMCs, and *in vivo* after balloon injury in rat aorta [21]–[23]. PPARγ agonists inhibit mRNA and protein expression, as well as induction of gelatinolytic activity of MMP-9 [24], an MMP implicated in SMC migration [25]. Here, we demonstrate that vanin-1 plays a major role in mediating both oxidative stress and PPARγ homeostasis in SMCs, as well as migration, proliferation, and MMP-9 activity. Furthermore, vanin-1 drives neointima formation after carotid artery ligation in mice.

## Results

### Expression of Vnn1 and Vnn3 and Pantetheinase Activity in SMCs

**Figure 1:**
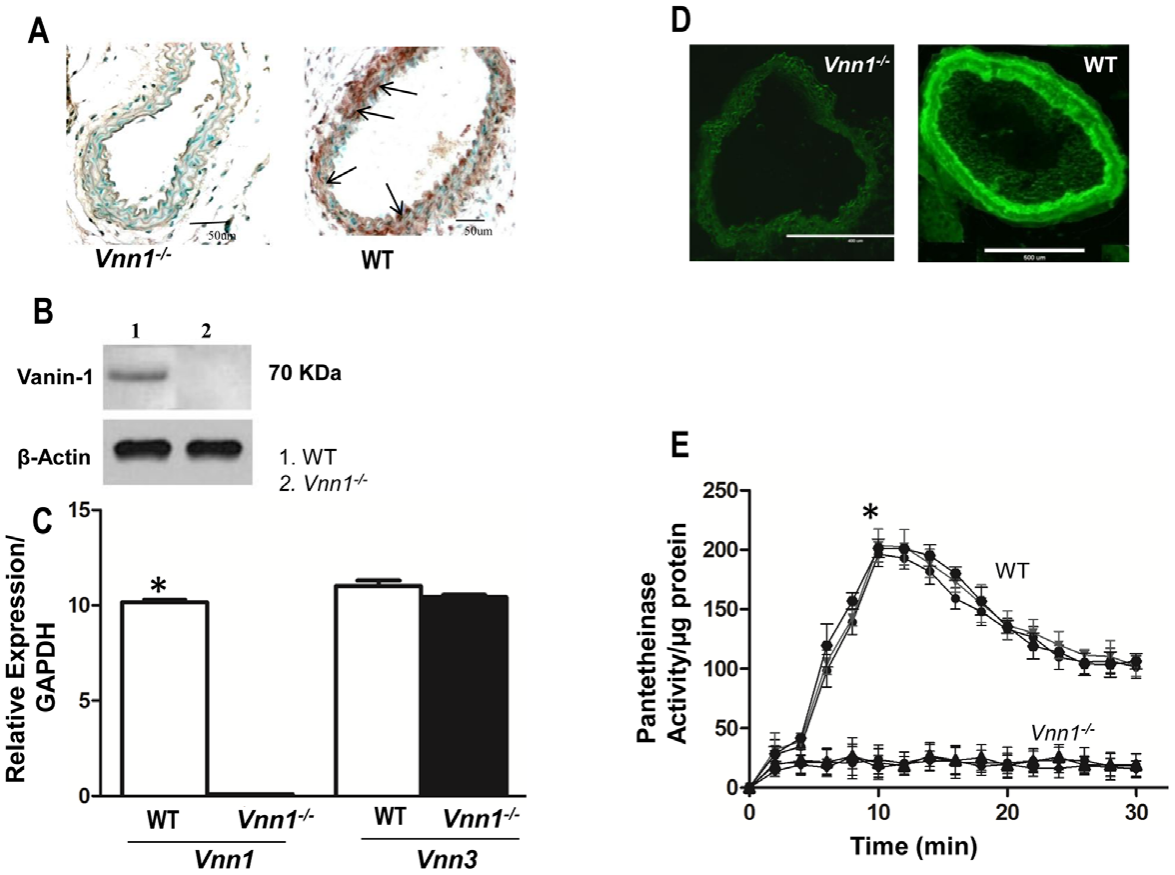
Constitutive vanin-1 expression in aorta and pantetheinase activity in cultured mouse aortic SMCs. *A* *,* Histologic sections of aortae from WT and *Vnn1^−/−^* mice were immunohistochemically stained for vanin-1 (brown positive staining). ***B***, Vanin-1 analyzed by aortic tissue Western blotting. ***C*** *,* In isolated aortic SMCs, vanin-1 and vanin-3 isoenzyme mRNA expression levels were compared by real-time PCR, normalized to GAPDH mRNA in samples from WT and *Vnn1^−/−^* mice. *P<0.05, WT vs. *Vnn1^−/−^* control. ***D*** *,* Aortic sections were incubated with the substrate pantothenate–AMC and constitutive vanin-1 immunofluorescence (green) is shown in carotid sections of WT and *Vnn1^−/−^* mice. ***E*** *,* Pantetheinase activity is shown from cultured SMC lysates of mice of indicated genotypes; results from SMCs of different animals are shown as distinct plots, with each result the mean ± SD of 3 independent experiments. *P<0.05 for WT vs. *Vnn1^−/−^.*

Vanin-1 was constitutively expressed in WT mouse aorta but not in *Vnn1^−/−^* mice aortae (Fig. 1A), a finding buttressed by aortic tissue Western blot analyses (Fig. 1B). The vanin enzyme subfamily includes two enzymes in mice (vanin-1, and secretory vanin- 3) [5], [8]. Quantitative PCR indicated that *Vnn1* deficiency was not associated with compensatory increase in the expression levels of *Vnn3* normalized to GAPDH (ie, vanin-3 expression relative to GAPDH not significantly different in WT vs. vanin-1 knockout SMCs (11.03±0.28 WT vs.10.43±0.121 *Vnn1^−/−^* SMCs) (Fig. 1C). Last, pantetheinase activity, demonstrated by fluorescence using the substrate pantothenate–AMC, was robust in WT mouse arterial sections and SMC lysates. However, the attenuation of detectable enzyme activity in artery sections or SMC lysates from *Vnn1^−/−^* mice indicated vanin-1 expression to be required for most arterial pantetheinase activity *in situ* (Fig. 1 D–E).

### A Vanin-1 Regulatory Circuit with GSH Mediates Oxidative Stress in SMCs

**Figure 2:**
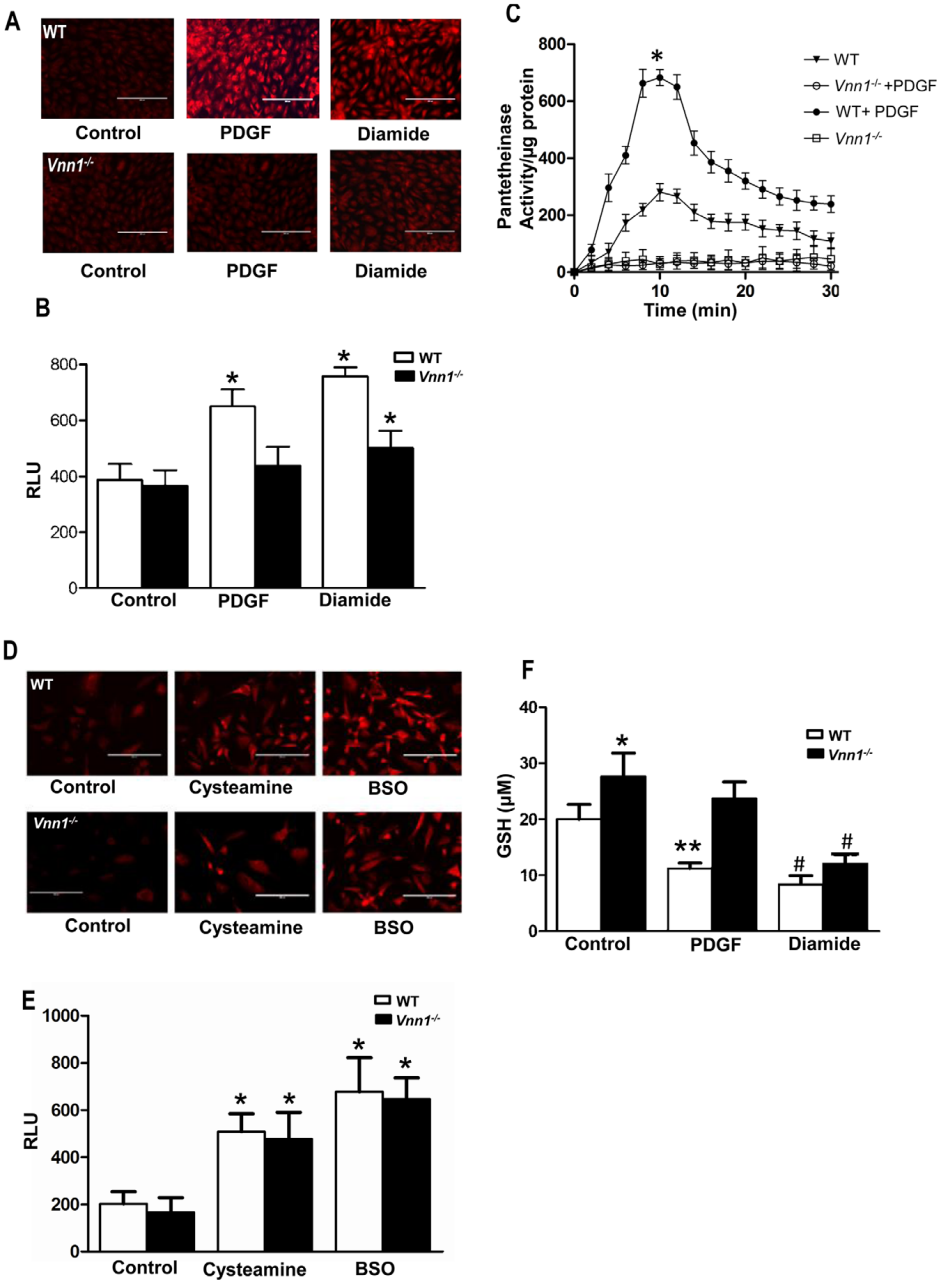
Vanin-1 and cysteamine modulate SMC superoxide production and GSH content in response to diamide and PDGF. *A*, SMCs isolated from *Vnn1^−/−^* and WT mouse aortae were growth-arrested in 0.1% calf serum for 24 h, and exposed to dihydroethidium (DHE) (10 µM). Images were captured 30 min after stimulation with diamide (5 µM) and PDGF (10 ng/ml). ***B*** *,* Superoxide production was quantified by flow cytometry (excitation and emission wavelengths 488 nm and 610 nm, respectively). ***C*** *,* WT and *Vnn1^−/−^* SMCs were treated with PDGF (10 ng/ml) for 24 h, and pantetheinase activity measured (*WT+PDGF vs. *Vnn1^−/−^* +PDGFand WT control). ***D*** *,* In SMCs treated with cysteamine (500 ng/ml) or BSO (1 µM) for 48 h, DHE fluorescence was measured. ***E*** *,* WT and *Vnn1^−/−^* SMCs were treated with PDGF (10 ng/ml) for 24 h, and GSH content measured via enzymatic recycling assay. Data are mean ± SD of 3 independent experiments. *P<0.05 vs. control; **control vs. PDGF in WT, # control vs. diamide in WT and *Vnn1^−/−^*. ***F*** *,* SMC superoxide production in response to cysteamine and BSO treatment was quantified by flow cytometry (excitation and emission wavelengths 488 nm and 610 nm, respectively). Data are mean ± SD of 3 independent experiments.

PDGF (10 ng/ml) and diamide (5 µM), a membrane-permeable thiol that oxidizes GSH, induced superoxide in WT SMCs; both these responses were blunted in *Vnn1^−/−^* SMCs, as assessed using the redox-sensitive dye Dihydroethidium (DHE) and by flow cytometry) (Fig. 2A–B). Next, we observed that PDGF treatment increased pantetheinase activity in WT but not in *Vnn1^−/−^* SMCs (Fig. 2C). Treatment with the vanin-1 enzymatic product cysteamine, a γGCS inhibitor, increased ROS levels in both WT and *Vnn1^−/−^* SMCs as did treatment with another GSH-depleting γGCS inhibitor buthionine sulfoximine (BSO; 1 µM) (Fig. 2 D,E). GSH levels in *Vnn1^−/−^* SMCs were significantly higher than in WT SMCs, with or without PDGF treatment (Fig. 2F). However, the GSH-oxidizing agent diamide reduced reduced GSH stores down to a comparable level in WT and *Vnn1^−/−^* SMCs (Fig. 2F). Therefore, we assessed for mechanisms beyond GSH depletion by which vanin-1 could modulate SMC function, and focused next on PPARγ.

### Vanin-1 Modulated PPARγ Expression Partly Regulates SMC Proliferation and Oxidative Stress

**Figure 3:**
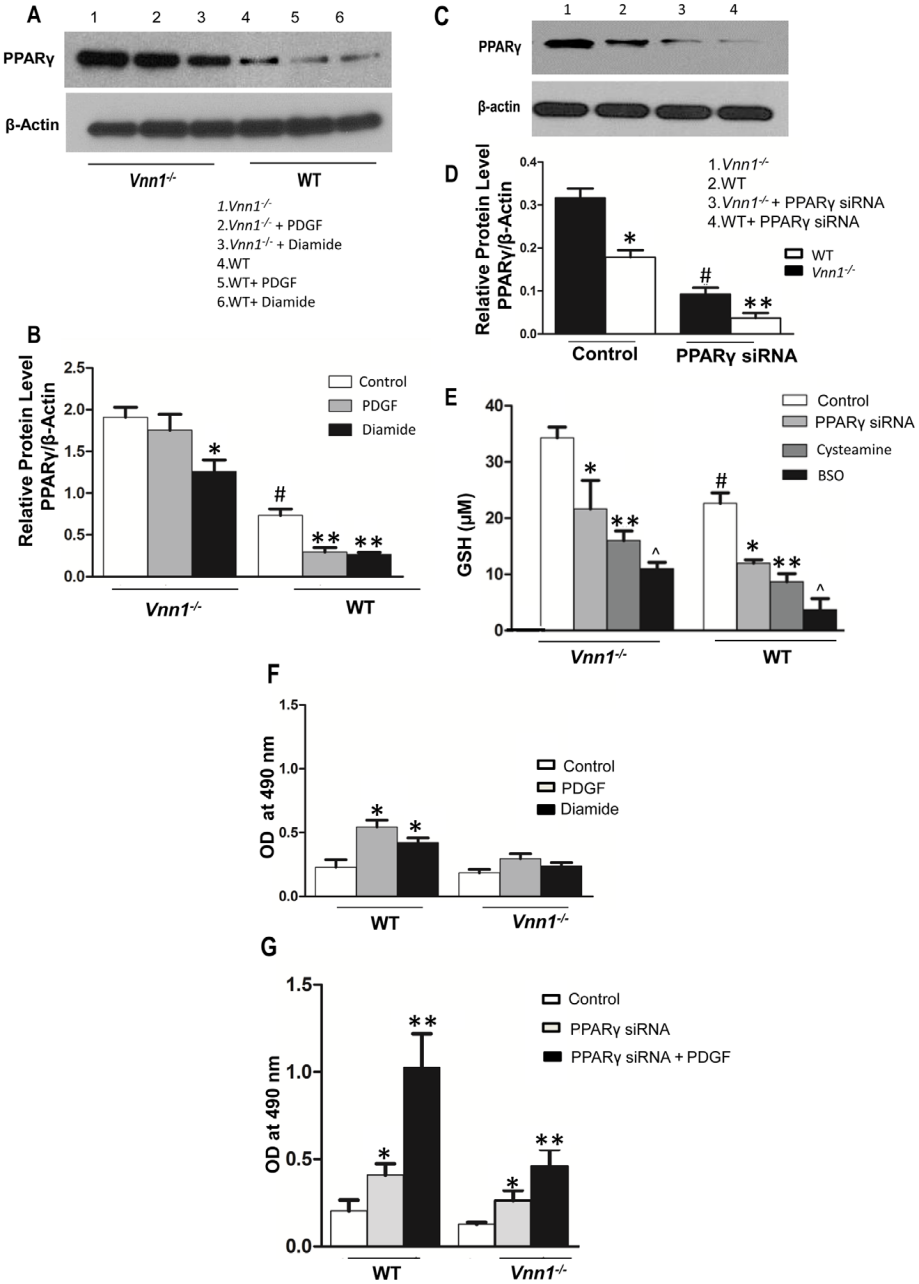
Relationships between PPARγ expression, GSH content, and vanin-1 in arteries and cultured SMCs. *A–B* *,* We treated cultured aortic SMCs with diamide (5 µM) and/or PDGF (10 ng/ml) for 48 h and PPARγ expression was analyzed by Western blot, and densitometry. P<0.05 for ^#^ *Vnn1^−/−^* control vs. WT control SMCs; *Vnn1^−/−^ control vs. diamide treated SMCs; **WT SMCs vs. PDGF and diamide treated SMCs, respectively. ***C–D*** *,* SMCs were transfected with PPARγ siRNA, and then PPARγ expression analyzed by Western blot. *P<0.05 for *Vnn1^−/−^* vs. WT SMCs, # *Vnn1^−/−^* control vs. PPARγ siRNA, **PPARγ siRNA WT SMCs. ***E*** *,* WT and *Vnn1^−/−^* SMCs were treated with cysteamine (500 ng/ml) or BSO (1 µM), and GSH content determined after deproteinization**.** Data pooled from 3 experiments done in triplicate. ^#^ P<0.05 *Vnn1^−/−^* vs. WT SMCs, *control vs. PPARγ siRNA,**control vs. cysteamine in both *Vnn1^−/−^* and WT SMCs, ^Λ^ control vs. BSO treatment in both *Vnn1^−/−^* and WT SMCs. In Panel ***F***, WT and *Vnn1^−/−^* SMCs were treated with diamide (5 µM) or PDGF 10 ng/ml) for 24 h, and cell proliferation compared. *P<0.05 vs. control. ***G*** *,* SMC proliferation using PPARγ siRNA knockdown. Data are mean ± SD of 3 independent experiments. *P<0.05 control vs. PPARγ siRNA, **PPARγ siRNA vs. PPARγ siRNA + PDGF in WT and *Vnn1^−/−^* SMCs.

PPARγ expression was constitutively elevated in both mouse aortic sections and cultured SMCs of *Vnn1^−/−^* mice compared to WT counterparts, as confirmed by Western blotting (Fig. 3A–B). Therefore, we assessed the inter-relationships between PPARγ, GSH, and vanin-1 in cultured SMCs, testing the potential contribution of vanin-1 modulation of PPARγ to changes in SMC proliferation and oxidative stress mediated by vanin-1. First, *Vnn1^−/−^* SMCs were relatively resistant to the capacity of diamide to reduce PPARγ expression (Fig. 3A–B). Moreover, under these conditions, PDGF treatment decreased PPARγ expression in WT SMCs but not in *Vnn1^−/−^* SMCs (Fig. 3B). In contrast, diamide significantly reduced PPARγ levels in WT SMCs compared to *Vnn1^−/−^* SMCs.

**Figure 4:**
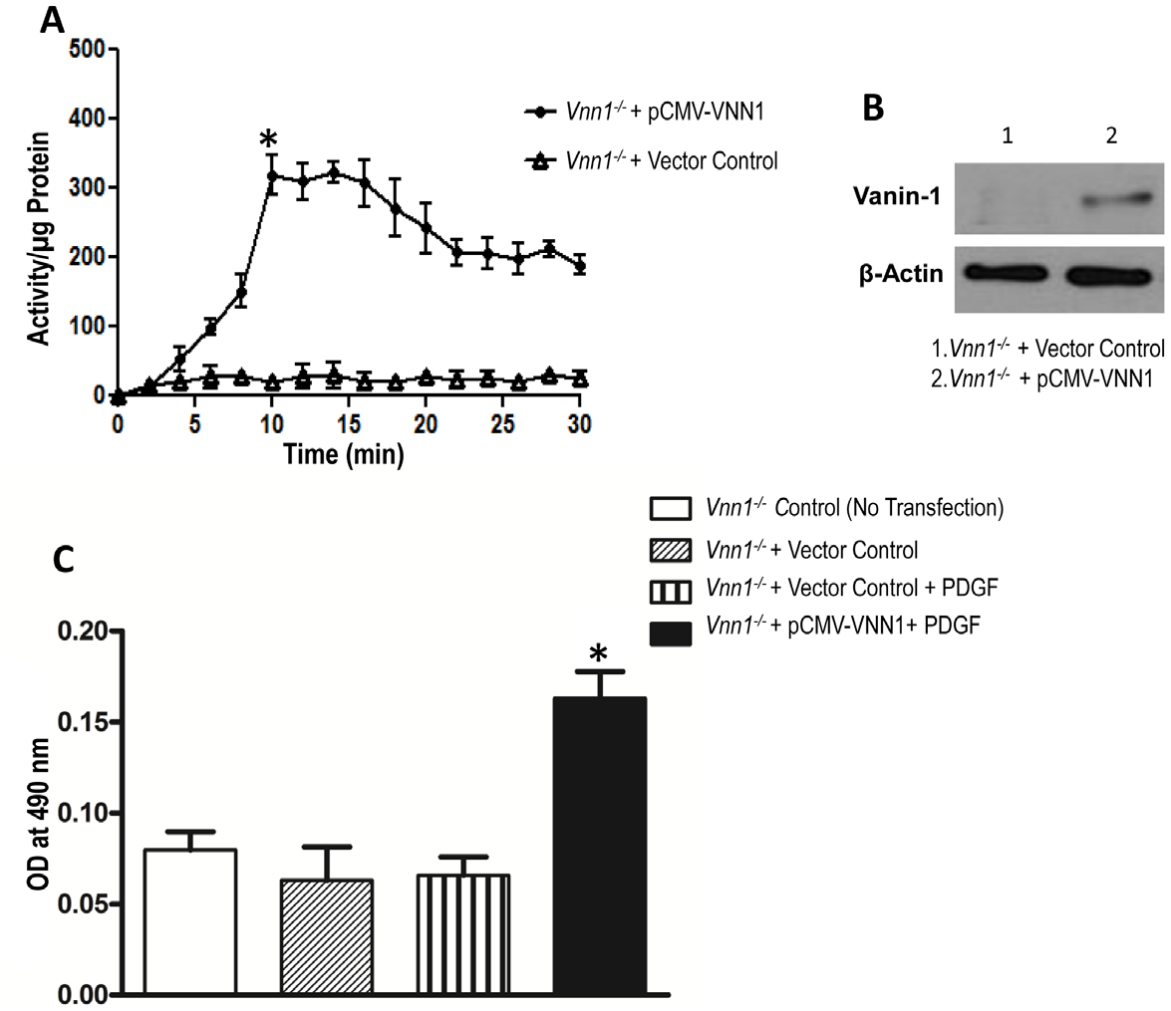
Vanin-1 mediates SMC proliferation in response to PDGF. Vanin-1 was expressed in *Vnn1^−/−^* SMCs by transfection using pCMV-VNN1 expression vector. ***A*** *,* Pantetheinase activity was measured in *Vnn1^−/−^* SMCs after transfection and ***B*** *,* Western blot analysis confirmed vanin-1 expression after transfection. ***C*** *,* Following pCMV-VNN1 or empty vector transfection, SMCs were treated with PDGF (10 ng/ml) for 24 h, and proliferation measured. Data are mean ± SD of 3 independent experiments. *P<0.05 *Vnn1^−/−^* SMCs control vs. *Vnn1^−/−^* + pCMV-VNN1+ PDGF.

Second, when we effectively knocked down PPARγ via siRNA (Fig. 3C,D), we observed decreased GSH levels in both WT and *Vnn1^−/−^* SMCs (Fig. 3E). Though this effect of PPARγ knockdown was not as extensive as the GSH depletion in response to treatment with cysteamine or BSO (Fig. 3E), it linked PPARγ expression with GSH homeostasis. Third, both PDGF and diamide increased SMC proliferation in WT SMCs, but *Vnn1^−/−^* SMCs were resistant to induction of proliferation by PDGF and diamide (Fig. 3F). Even when PPARγ was knocked down, PDGF induced proliferation more in WT than *Vnn1^−/−^* SMCs (Fig. 3G). Similarly, *Vnn1^−/−^* SMCs also were more resistant to the capacity of the PPARγ inhibitor GW9662 to promote SMC proliferation (data not shown). Fourth, we expressed human vanin-1 by transfection in *Vnn1^−/−^* SMCs and linked increased pantetheinase activity and vanin-1 (Fig. 4A–B) with a permissive state for SMC proliferation to be induced by PDGF (Fig. 4C). Taken together, vanin-1 induced oxidative stress and enhanced SMC proliferation, doing so only partially by affecting PPARγ expression in SMCs. Conversely, PPARγ expression modulated sensitivity of SMC proliferation in response to oxidative stress.

### Vanin-1 Also Modulates SMC MMP Activity and Migration

**Figure 5:**
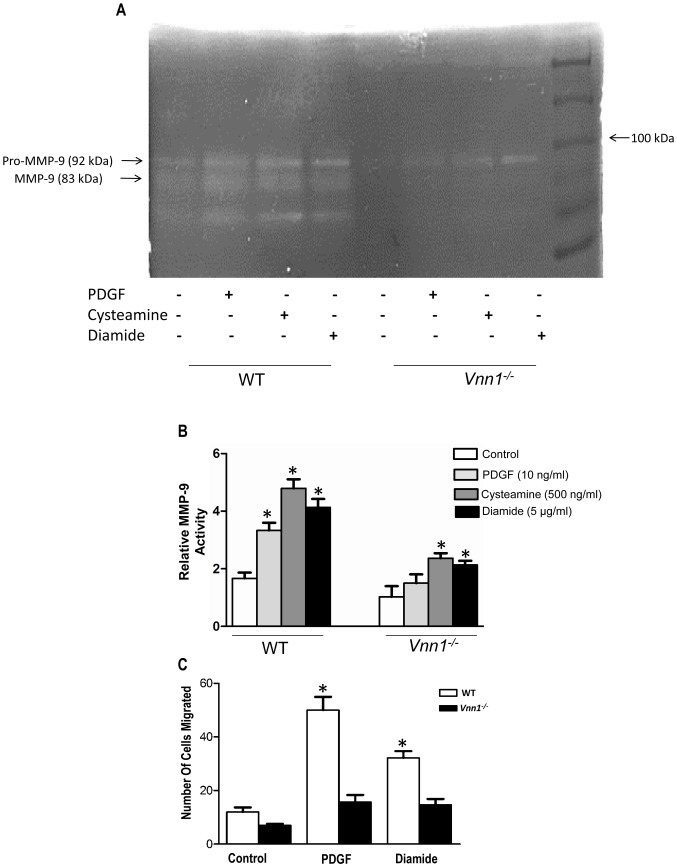
Activation of MMP-9 and migration are inhibited in *Vnn1* *^−/−^* **SMCs. ** ***A*** *,* Serum-starved SMCs were treated with PDGF (10 ng/ml), cysteamine (500 ng/ml) or diamide (5 µM) for 48 h, and conditioned media analyzed for MMP-9 activity by gelatin zymography. ***B*** *,* Densitometric analysis of enhanced MMP-9 activity in WT compared to *Vnn1^−/−^* SMCs. Data pooled from 3 independent experiments for densitometry. ***C*** *,* We assayed migration in SMCs treated with diamide (5 µM) or PDGF (10 ng/ml), as described in the Methods. Total number of SMCs migrated/well after 48 h treatment with diamide or PDGF are shown. Data are mean ± SD of 3 independent experiments. *P<0.05 vs. control.

Diamide and PDGF, as well as cysteamine, induced MMP-9 activity more in WT than *Vnn1^−/−^* SMCs (Fig. 5A–B). In addition, vanin-1 deficiency significantly decreased both diamide-induced and PDGF-induced migration of cultured SMCs (Fig. 5C). Given the collective findings on SMC proliferation, oxidative stress, MMP activity, and migration in vanin-1 deficient SMCs, we concluded the studies by examining the role of vanin-1 in arterial remodeling and PPARγ expression in response to carotid artery ligation *in situ*.

### Vanin-1 Deficiency Inhibits Post-injury Carotid Artery Neointimal Hyperplasia

**Figure 6:**
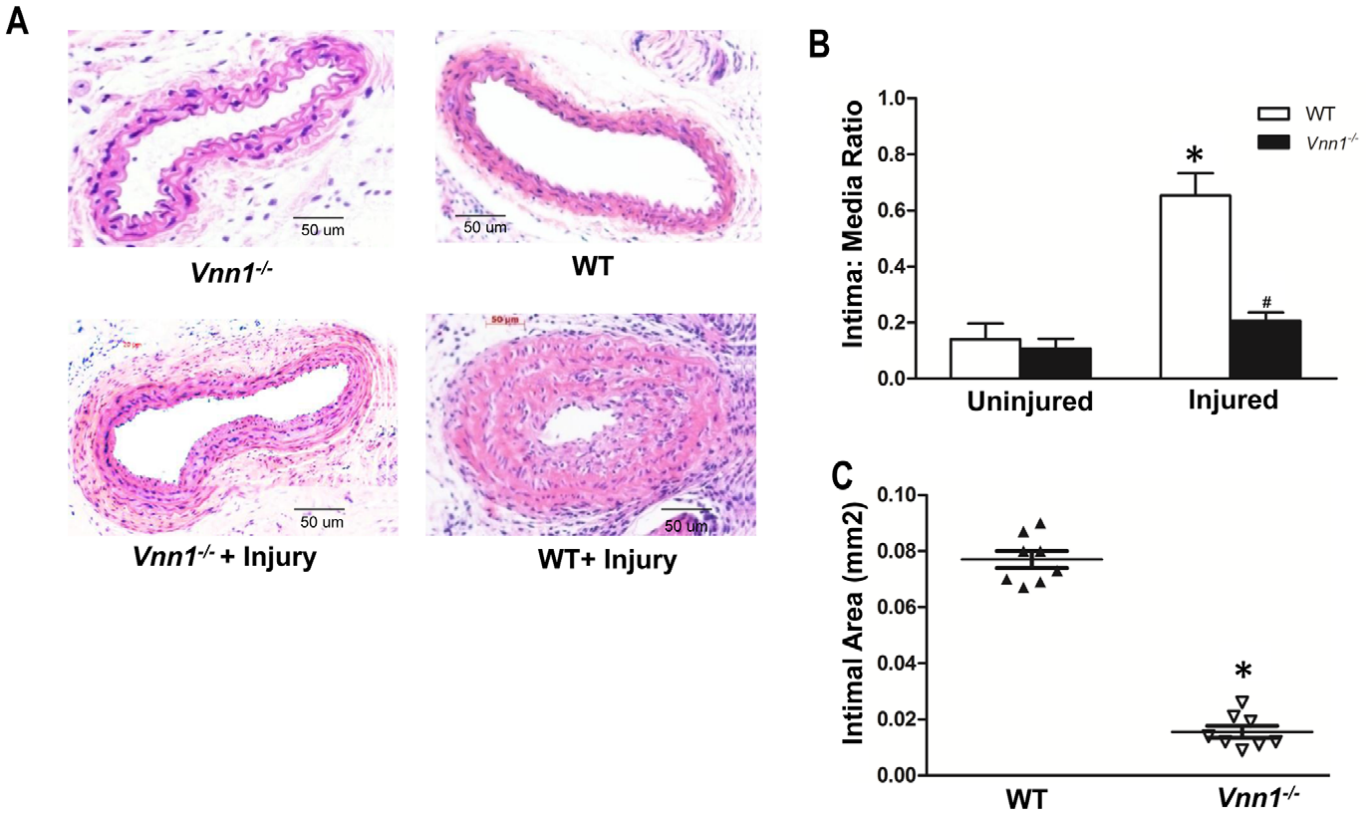
Reduced neointima formation after carotid ligation in *Vnn1* *^−^* ^*/−*^ mice. ***A*** *,* Cross sections of representative H&E stained carotid arteries of *Vnn1^−/−^* and WT mice are shown, 21 days after carotid ligation**.** ***B–C*** *,* For each arterial section, intimal and medial areas were measured, and intima:media ratio calculated. Morphometric analysis was performed using NIH Image Analysis Software. Data are mean ± SD of 3 independent experiments *P<0.05 WT vs. *Vnn1^−/−^*.

**Figure 7:**
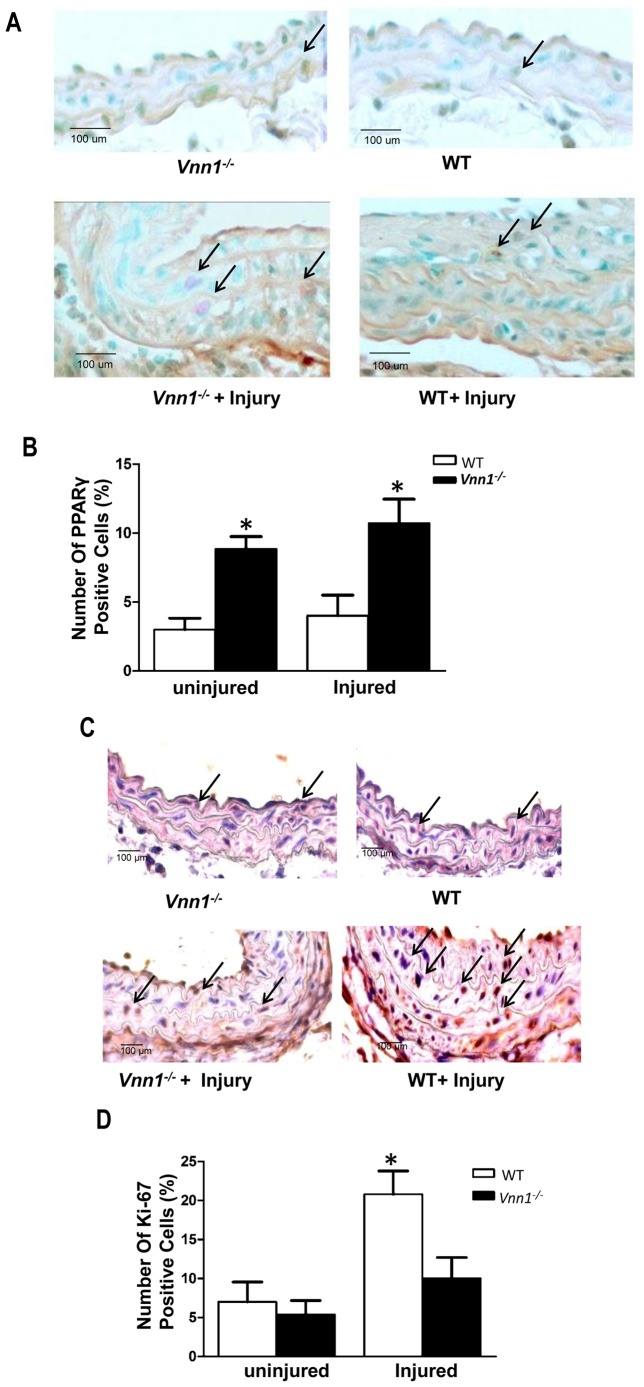
Immunohistochemical staining for PPARγ and the cell proliferation marker Ki-67 in uninjured and injured carotid arteries. *A* *,* Paraffin-embedded, uninjured and injured aortae of the indicated genotypes were cut into 6 µm sections for immunohistochemistry, with PPARγ positive media and neointimal cells staining dark brown (arrowheads) in methyl green counterstained sections. ***B*** *,* Percentage of arterial media and neointimal PPARγ positive cells, determined as described in Methods. ***C*** *,* Immunohistochemical detection of Ki-67 in representative WT and *Vnn1^−/−^* carotid sections. ***D*** *,* Percentage of arterial media and neointimal Ki-67 positive cells. Data are mean ± SD of 3 independent experiments. *P<0.05 WT vs. *Vnn1^−/−^*.

We observed robust development of neointima in WT mice following left carotid artery ligation, but this vascular remodeling injury response was attenuated in *Vnn1^−/−^* mice (Fig. 6A). Specifically, injured carotid arteries of *Vnn1^−/−^* mice displayed markedly decreased intima:media ratio (Fig. 6B) and cross sectional area of the neointima (Fig. 6C). There was more robust PPARγ expression in injured *Vnn1^−/−^* arteries compared to WT arteries (Fig. 7A–B). Last, we observed decreased cell proliferation, assayed by Ki-67 staining, in both the media and neointima in the injured *Vnn1^−/−^* mouse arteries (Fig. 7C–D).

## Discussion

Oxidative stress, including NADPH oxidase activity [2], [26]–[30], and regulation of PPARγ [25], are among the numerous factors implicated in activation of SMCs in vascular remodeling [21]. Given putatively redundant pathways for vascular remodeling, the net individual roles of GSH stores and PPARγ in the process, let alone potential impact of their combined role, had not previously been clear. This study identified vanin-1 as a central mediator of oxidative stress and an inhibitor of constitutive PPARγ expression in SMCs. Vanin-1 promoted GSH depletion and MMP activation, and vanin-1 critically mediated PDGF-induced proliferation and migration in cultured SMCs. The vanin-1 enzymatic product cysteamine also promoted oxidative stress, MMP activity, and decreased PPARγ expression in SMCs. These findings were buttressed by the demonstration that vanin-1 knockout markedly limited *in vivo* post-injury neointima formation. Moreover, vanin-1 deficiency resulted in reduced lesion cell proliferation and lesion PPARγ expression relative to ligated WT control carotid arteries.

In our *in vitro* studies, SMCs from *Vnn1^−/−^* mice failed to demonstrate increased migration in response to PDGF and diamide. We also observed that regardless of whether diamide or PDGF was promoting oxidative stress, vanin-1 played a crucial role in regulating generation of ROS. Diamide acts by formation of a protein-protein internal disulfide bond, without formation of a sulfenic acid intermediate [31]. PDGF-BB-induced AP-1 activity and cell proliferation are secondary to alkylation of cysteinyl residues, essential for the catalytic activities of various enzymes, transcription factors and/or transporters [32], and PDGF effects in SMCs were blocked by addition of catalase or antioxidants in prior studies [4]. It is noteworthy that SMCs express multiple enzymes that generate ROS, including phospholipases, cytochrome P450, cyclooxygenase, lipoxygenase, xanthine oxidase, and ribonucleotide reductase, as well as a functional NADPH oxidase complex 2630. Moreover, vascular NADPH oxidase and ROS mediate functionally significant signal transduction in SMCs [26]–[30]. Vanin-1 is likely active in regulating SMC function because cellular GSH homeostasis modulates not only oxidative stress but also pro-inflammatory sequelae [26]. In this context, GSH depletion is one mode for enhancement of inflammation signaling pathways such as p21*ras*, MAP kinase activity, and NF-κB nuclear translocation [26], [33]. Our demonstration of a major role of vanin-1 in depleting SMC GSH stores identifies a novel pro-inflammatory activation switch in SMCs. In this study, siRNA-induced silencing of PPARγ, by itself, depleted SMC GSH levels and modulated the threshold for SMC activation in response to oxidative stress. Our results, for SMCs, treated with PPARγ siRNA and pharmacologic PPARγ inhibition, indicated that vanin-1 enhanced SMC activation responses in part by modulation of PPARγ expression. PPARγ exerts substantial effects on SMC proliferation *in vitro* and *in vivo* [19], [20], [34]–[36]. In addition, PPARγ activation is an SMC anti-inflammatory control point that inhibits activation of NF-κB, and suppresses SMC proliferation induced by PDGF and angiotensin II [19], [20], [34]–[36]. PPARγ activation also inhibits SMC invasiveness, and migration mediated by MMP activation [20]. Multiple overlapping mechanisms regulate PPARγ expression in SMCs [19], [20], [35]. Importantly, lack of vanin-1 robustly inhibited PPARγ expression in SMCs, and, conversely, PPARγ expression *in situ* was increased constitutively, and after carotid arterial injury, in *Vnn1^−/−^* mouse arteries.

Limitations of the current study include confinement of the scope of *in vivo* analyses to carotid artery ligation, and the inherent limits of *in vitro* experiments in SMCs. It remains to be determined what effects vanin-1 has in endothelial cells, macrophages, and in other resident and infiltrating cell types in the artery wall that modulate vascular remodeling. Vanin-1 pantetheinase effects on individual elements in ROS generation, such as NADPH oxidase activity and mitochondrial activities, merit further investigation. We also restricted our analyses on PPAR to PPARγ. Net effects of vanin-1 on individual PPARγ isoforms, let alone potential effects on other PPARs that affect the artery [35], remain to be investigated. Oxidative stress is difficult to target in arteries and other tissues, since multiple mechanisms contribute to reactive oxygen species generation and elimination [26]–[30]. Moreover, PPARγ activity has been difficult to target for vascular and other disease in clinical medicine, since PPARγ activating thiazolidinediones (TZD) drugs can cause side effects, including severe and potentially lethal fluid retention mediated by renal effects of PPARγ activation [37]. In this context, it is noteworthy that vanin-1 knockout mice are viable and grossly normal. Our study reveals vanin-1 to be a novel inflammatory switch for vascular remodeling diseases, via dual effects on PPARγ and oxidative stress.

## Methods

### Materials

Diamide (1,1'-azobis(*N*,*N*-dimethylformamide)), PDGF-BB, and all chemical reagents were obtained from Sigma-Aldrich (St. Louis, MO), unless otherwise indicated. Antibodies against β-actin and Vanin-1 were from Cell Signaling (Boston, MA) and Santa Cruz Biotechnology, Inc (Santa Cruz, CA) respectively. β-alanine 7-amido-4-methylcoumarin trifluoroacetic acid was from Chem-Impex International Inc (Wood Dale, IL). The GSH assay kit was from Cayman Chemicals (Ann Arbor, MI) and the cell proliferation assay kit was from Promega (Madison, WI). DHE was from Molecular Probes (Grand Island, NY). Anti-Ki-67 antibody was from Abcam (Kendall Square, Cambridge, MA).

### Mice Studied

All animal procedures were performed humanely and followed institutionally approved protocols, with procedures in compliance with the standards for care and use of laboratory animals of the Institute of Laboratory Animal Resource. *Vnn1^+/−^* mice were backcrossed for more than nine generations on a C57BL/6 background, and then interbred to generate and study *Vnn1^−/−^* mice and wild-type littermate progeny on the same background, as described [10], [38]. All animal experimentation was assessed and approved by the IACUC (Institutional Animal Care and Use Committee) of the San Diego Veterans Affairs Medical Center.

### Carotid Artery Ligation

Animals were anesthetized by intraperitoneal (i.p.) injection of 15 mg/kg Ketamine (phoenix pharmaceutical inc, ST. Joseph, MO), 1.6 mg/kg Xylazine (Akorn Inc, Decatur, IL), and 1.2 mg/kg Acepromazine (Boehringer Ingelheim, Ridgefield, CT). In brief, the left common carotid artery, dissected from surrounding connective tissue, was ligated through a midline neck incision just proximal to its bifurcation, using 6–0 silk ligature. In control groups, dissection of the left common carotid artery from the surrounding connective tissue was performed without ligation. Twenty-one days after injury or simple dissection, animals were anaesthetized and perfused with PBS, followed by 4% paraformaldehyde. Carotid arteries were excised, and then embedded in paraffin. Cross-sections (6 microns) were taken starting at the ligation site and stained with hematoxylin and eosin.

### SMC Culture and Transfection

Mouse aortic SMCs were isolated by enzymatic digestion [39] from *Vnn1^−/−^* and control littermate WT mice. Cells were cultured in DMEM supplemented with 10% FBS, 100 Units/ml penicillin, 100 µg/ml streptomycin, and 2 mM L-glutamine at 37°C in a humidified 95% air and 5% CO2 incubator. The purity of each mouse SMC preparation in culture was confirmed by immunocytochemistry for α-smooth muscle actin. Cells were passaged at 1∶3 ratio. Experiments were performed using cells between passage 3–8, and serum-deprived conditions were generated by incubation for 24 h in DMEM containing 0.1% FBS. The small interfering RNA (siRNA) and scrambled RNA (scRNA) employed were purchased from Santa Cruz Biotechnology (Santa Cruz Biotechnology, Inc. Santa Cruz, CA). Transfection was done by manufacturer recommended protocol. *Vnn1^−/−^* SMCs were transfected with pCMV-VNN1 full-length plasmid DNA using 293T transient overexpression reagent (Abnova, Walnut, CA, Catalog # H00008876-T01), as per manufacturer instructions.

### Quantitative Real-time RT-PCR

Total RNA was isolated using Trizol (Invitrogen, San Diego, CA), and cDNA was transcribed from 1 µg of RNA using Superscript III enzyme (Invitrogen), and random primers. Reverse transcription and quantitative real-time RT-PCR were performed using the LightCycler 2.0 (Roche Diagnostics, San Francisco, CA), as described [38]. Oligonucleotides were designed for mouse vanin-1, using Roche Primer Express software: vanin-1, forward 5'-TGGTAGTTCAGTGGACACG-3', and reverse 5'- AGGGAAGACATACCGGG-3', and mouse vanin-3, 5-CCGTTTGGGAAGTTTGGC-3' and reverse 5'-CGAATGGAATGGAACTGCTGA-3'. Following amplification, a monocolor relative quantification of the target gene and reference (glyceraldehyde-3- phosphate dehydrogenase; (GAPDH, F 5′-CATCCCAGAGCTGAACG-3′, R 5′- CTGGTCCTCAGTGTAGCC-3′) was normalized for target gene to GAPDH mRNA (glyceraldehyde-3-phosphate) copy ratios using the manufacturer’s LightCycler software (version 4.0).

### SDS-PAGE and Western Blotting

Aortic tissue and SMCs were lysed in RIPA buffer (50 mM Tris-HCl [pH 7.4], 150 mM NaCl, 1% NP-40, 0.5% sodium deoxycholate, 0.1% SDS, 10 mM sodium fluoride,1 mMsodium orthovandate, 1 mM phenylmethylsulfonyl fluoride, 1 mM EDTA, 10 µg/ml leupeptin, 1 µg/ml aprotinin) and protein extracts separated by 10% SDS-PAGE, and transferred to Immobilon-P membranes. After blocking, blots were incubated with primary antibodies overnight at 4°C, and detection of antigen using secondary antibody conjugated with horseradish peroxidase, and enhanced chemiluminescence, as well as scanning densitometry, were done as described [38].

### Pantetheinase Activity Assay

The substrate, pantothenate–AMC was chemically synthesized using β-alanine 7- amido-4-methylcoumarin trifluoroacetic acid (TFA) salt (H- β -Ala-AMC.TFA, 36 mg, 1eq) and R-(-)- pantolactone [40]. To assay pantetheinase activity, we used pantothenate–AMC as substrate at 37°C for 30 min, with the hydrolysis catalyzed by vanin-1 yielding pantothenic acid and detectable free fluorescent AMC (excitation 340 nm, emission 460 nm) [40]. Cells were washed three times with PBS and lysed with potassium phosphate buffer (100 mM, pH 7.5) containing 0.1% Triton X-100 and 0.6% sulfosalicylic acid. Protein concentrations were determined by bicinchoninic acid (BCA) protein assay. Enzymatic assay was carried out using 5 µg of cell lysate in phosphate buffer (100 mM potassium phosphate buffer, pH 7.5) containing 2 µM pantothenate- AMC, 0.01% BSA, 0.5 mM DTT, 5% DMSO, and 0.0025% Brij-35 in a total volume of 100 µl reaction mixture. Reactions were carried at 37°C in the presence or absence of cell lysate and the fluorescence (Excitation 350 nm and emission 460 nm) was recorded every 2 min, with the change in fluorescence measured over a 30 min period. A standard curve was generated using purified recombinant vanin-1 (Abnova, Walnut, CA, catalog # H00008876-P01) under the same buffer conditions described above. Vanin-1 activity was normalized for total protein content. Pantetheinase activity was calculated by taking the slope at 30 min, fitting the data to the standard curve and normalizing for total protein content.

### Quantification of GSH

To determine GSH and oxidized glutathione levels, we used an enzymatic recycling assay (glutathione assay kit, Cayman Chemicals, Ann Arbor, MI) in the presence of glutathione reductase. We spectrophotometrically determined 5-thio-2-nitrobenzoic acid generation in deproteinated cells [38].

### Measurement of Superoxide Generation

Superoxide generation in SMCs was measured, as described [41], in cells incubated with dihydroethidium (DHE) (10 µM) in PBS at 37°C in the dark for 30 min in a 5% CO2 humidified chamber. Propidium iodide (10 µg/ml) was added 1 min before flow cytometry and examined by FACS with excitation at 488 and emission at 610 nm. Flow cytometry (FACScan; BD Biosciences, San Jose, CA) was used to select a homogeneous population of 10,000 live cells. Bivariate flow cytometry was performed with a FACS scan, and the data were analyzed with Cell Quest software (Becton Dickinson, San Jose, CA, USA), in the cell population from which apoptotic cells were gated out against forward and side scatter or PI-positivity. The geometrical mean of ethidium fluorescence intensity (excitation 488 and emission at 610 nm) in thepopulation was used for analysis. Alternatively, samples were examined by fluorescence microscopy (Advanced Microscopy Group, EVOS FL).

### Immunohistochemistry

Formalin-fixed and paraffin-embedded tissue sections were deparaffinized and followed by rehydration, endogenous peroxidase activity was quenched using 3% H2O2. Followed by blocking, the sections were incubated overnight at 4°C with primary antibody against vanin-1, PPARγ (1∶100) and Ki-67 (1∶100) as a cell proliferation marker, sections were then incubated with biotinylated secondary antibody and peroxidase-labeled (Invitrogen, Carlsbad, CA). Peroxidase activity was demonstrated by exposing sections to the substrate, 3,3′–diaminobenzidine tetrahydrochloride (DAB) and counterstaining with 1% methyl green. For negative control sections, PBS was substituted for the primary antibody. We calculated percentage of positive staining SMCs in media and neointima (relative to total cell number) in 5 different 20 X magnification fields in each section, studying 8 sections from each control and ligated sample from each mouse.

### SDS-PAGE/Western Blot Analysis

SMCs were lysed in RIPA buffer (50 mM Tris-HCl [pH 7.4], 150 mM NaCl, 1% NP-40, 0.5% sodium deoxycholate, 0.1% SDS, 10 mM sodium fluoride, 1 mM sodium orthovanadate, 1 mM phenylmethylsulfonyl fluoride, 1 mM EDTA, 10 µg/ml leupeptin, 1 µg/ml aprotinin). Protein extracts (35 µg) were separated by 10% SDS-PAGE, and transferred to Immobilon-P membranes, and, after blocking, blots were incubated with primary antibodies for 18 h at 4°C. The membrane was washed, and then incubated withrespective secondary antibody conjugated with horseradish peroxidase. Enhanced chemiluminescence reagent (Thermo Scientific, Rockford, IL,) served as substrate solution, used according to manufacturer instructions. Horizontal scanning densitometry of films was performed using universal hood densitometry (Bio-Rad, Hercules, CA).

### Assay of MMP Activity

Gelatinase activity was determined by zymography as previously described [42], [43]. Equal amounts of conditioned media from identical numbers of cells, grown under serum free conditions for 48 h, were loaded onto 10% SDS-polyacrylamide gels containing 0.1% gelatin, and zymography was performed. After electrophoresis, gels were incubated for 45 min in renaturation buffer and in developing buffer (Invitrogen, Carlsbad, CA) for 24 h at 37°C. Gels were stained with Coomassie blue; clear bands indicated active enzymes (MMP-9 pro-form, 92 kDa; active form, 83 kDa). Enzymatic activity was estimated by densitometry of negative-image zymographic gels and measured in OD units.

### Cell Proliferation and Migration Studies

We used the Invitrogen non-radioactive Cell Proliferation Assay kit, per manufacturer instructions, to determine cell proliferation. SMC migration was assayed using the Transwell system (Corning, NY), with a polycarbonate membrane in six-well plates. SMCs were plated at a concentration of 1.0x 10^6^ cells/ml. Numbers of cells migrated per well, after 48 h treatment with PDGF or diamide, were determined by thiozolidine bluestaining. Images were analyzed using a microscope with digital camera (Advanced Microscopy Group, Bothell, WA).

### Statistical Analyses

Unless otherwise indicated, data are presented as the mean ± SD of determinations from 3 or more experiments. Results were compared by one-way ANOVA followed by Bonferroni multiple-comparison analysis. A value of p<0.05 was used to define statistical significance.
